# Supplementary material for: The self-inhibitory nature of metabolic networks and its alleviation through compartmentalization
Source: Nat Commun. 2017 Jul 10;8:16018. doi: 10.1038/ncomms16018 (PMC5508129; doi:10.1038/ncomms16018)
Supplement: Supplementary Information [file ncomms16018-s1.pdf]

Type of file: PDF
Size of file: 0 KB
Title of file for HTML: Supplementary Information
Description: Supplementary figures, supplementary tables and supplementary references.

Type of file: XLSX
Size of file: 0 KB
Title of file for HTML: Supplementary Data 1
Description: This file contains the detailed information of the enzyme-inhibition network in seven spreadsheets. (1) Inhibition Network: Enzymes and Inhibitors with functional group information, (2) Inhibitors: Detailed information of all inhibitors of the inhibition network, (3) Enzymes: Detailed information of all enzymes of the inhibition network, (4) Competitive inhibition: structural similarities between substrates and inhibitors, (5) Noncompetitive inhibition: structural similarities between substrates and inhibitors, (6) Uncompetitive inhibition: structural similarities between substrates and inhibitors, (7) Pathways: Detailed information of pathways including enzyme, metabolites and inhibitors of enzymes of each pathway

Type of file: PDF
Size of file: 0 KB
Title of file for HTML: Peer review file
Description:

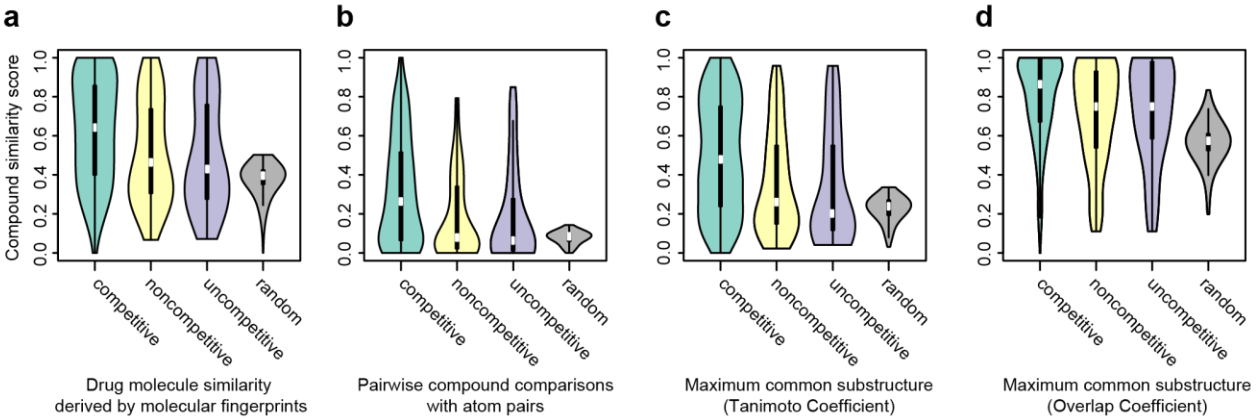

Supplementary Figure 1. Pairwise compound similarity between inhibitor and substrate for competitive, noncompetitive and uncompetitive type of inhibition, compared to similarity between pairs of random compounds, using the following computational similarity coefficients: (a) Drug molecule similarity derived by molecular fingerprints (calcDrugFPSim [1]), (b) Pairwise compound comparisons with atom pairs (cmp.similarity [2]), (c) Tanimoto Coefficient for Maximum common substructure (fmcsR [3]) (d) Overlap Coefficient Maximum common substructure [3].

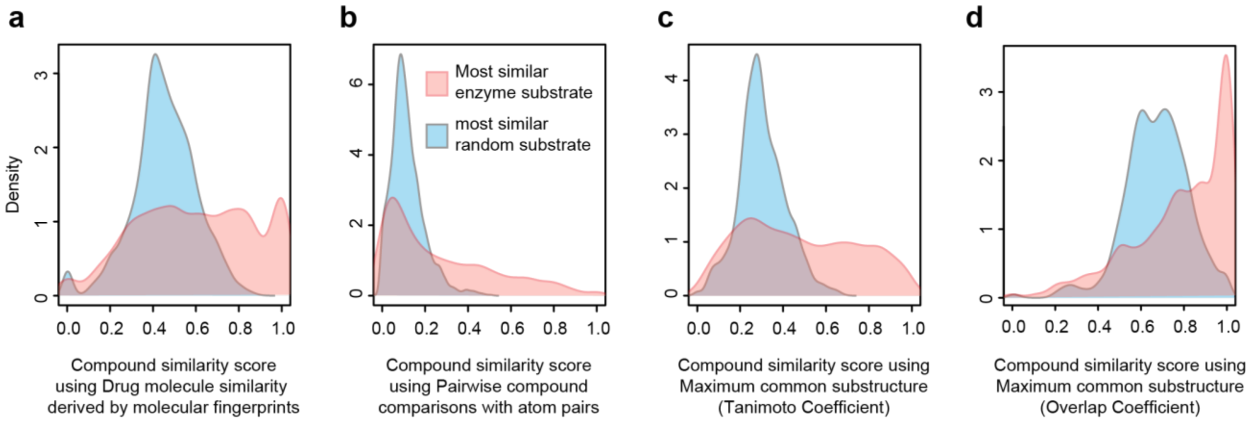

Supplementary Figure 2. Pairwise compound similarity between an inhibitor and the most similar substrate of an enzyme, created from all metabolites of an of enzyme. The similarity coefficients between metabolite-inhibitor have wide distribution up to full similarity, while the similarity between an inhibitor and the most similar random compound from a set of random substrates of similar size to the metabolites, has much lower similarity. Similarities given are (a) Drug molecule similarity derived by molecular fingerprints (calcDrugFPSim [1]), (b) Pairwise compound comparisons with atom pairs (cmp.similarity [4]), (c) Tanimoto Coefficient for Maximum common substructure [3] (d) Overlap Coefficient Maximum common substructure [3].

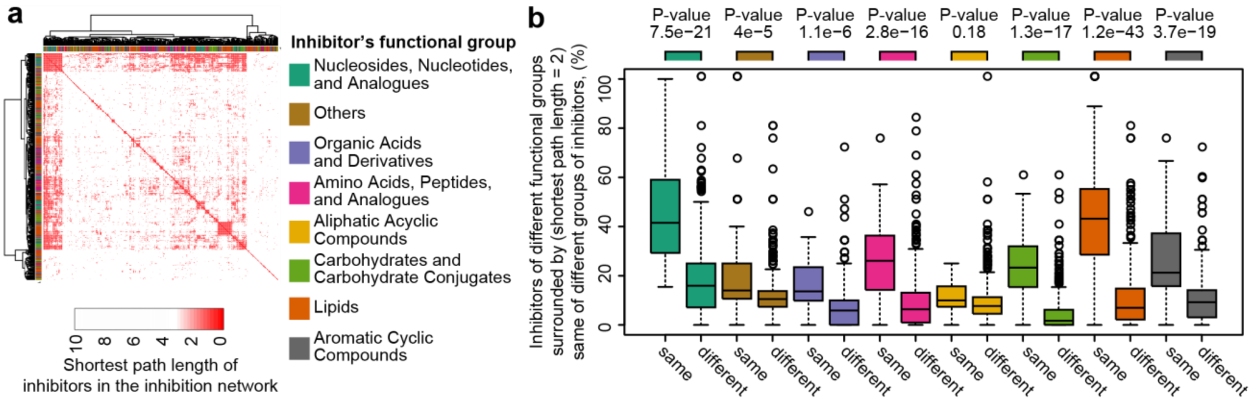

Supplementary Figure 3 (a) Shortest path length between all pair of inhibitors within the enzyme-inhibition network. (b) Closest inhibitors (shortest path length 2) within the enzyme-inhibition network are significantly likely to be from the same functional group (HMDB metabolite superclass) compared to inhibitors from the other functional groups. Enzyme inhibitors acting in trans are hence most likely functionally related to the enzymes metabolites. P-value calculated using Welch Two Sample t-test.

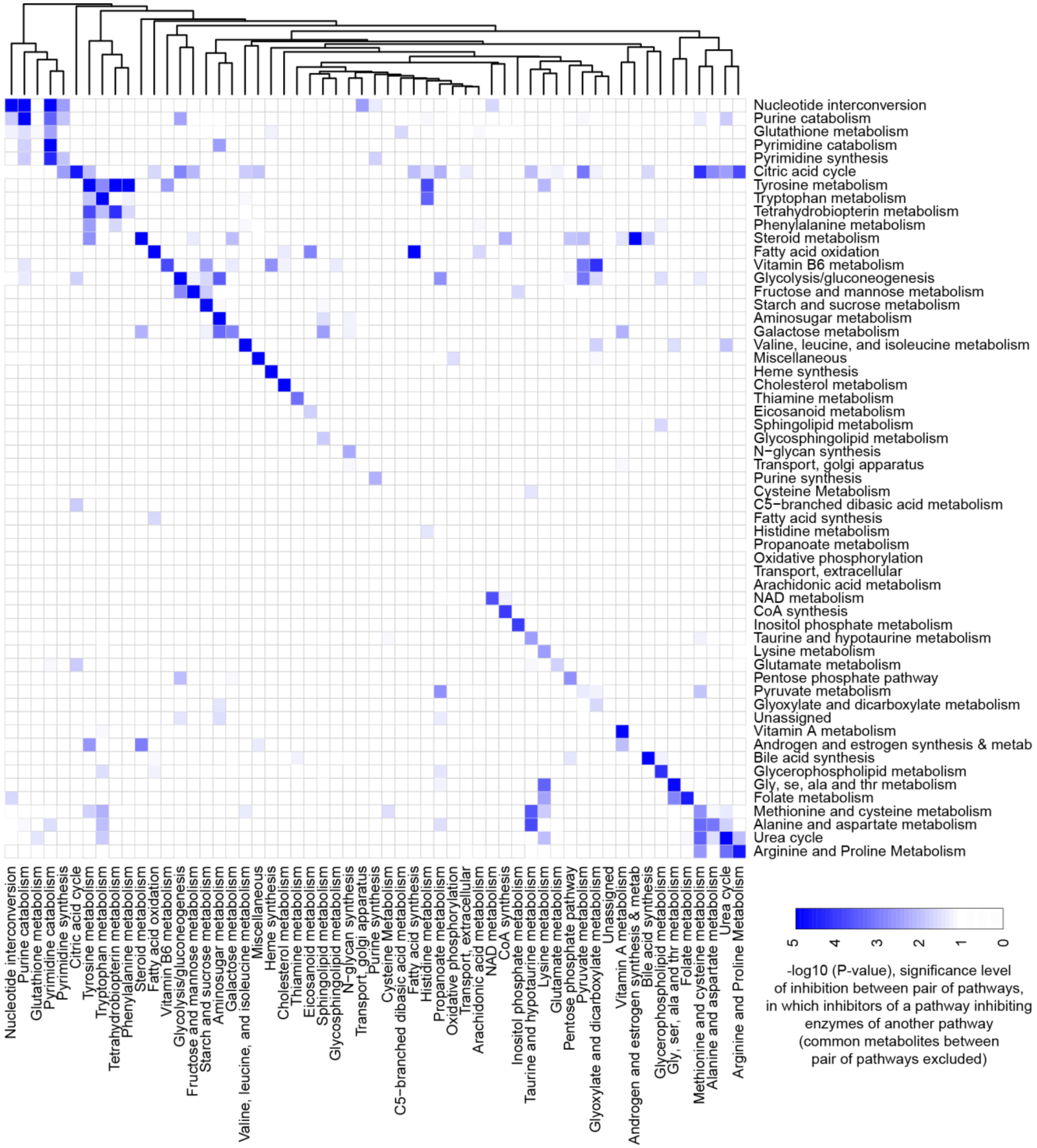

Supplementary Figure 4 Significance level of metabolic pathways inhibiting other pathways *in trans*. Common compounds shared between the pathways are excluded. Significance in the diagonal shows that inhibition within the same pathway is the most significant on the genomic level. As closer two metabolic pathways are in the topological structure of the metabolic network, the more likely they inhibit each other. P-value calculated using hypergeometric test.

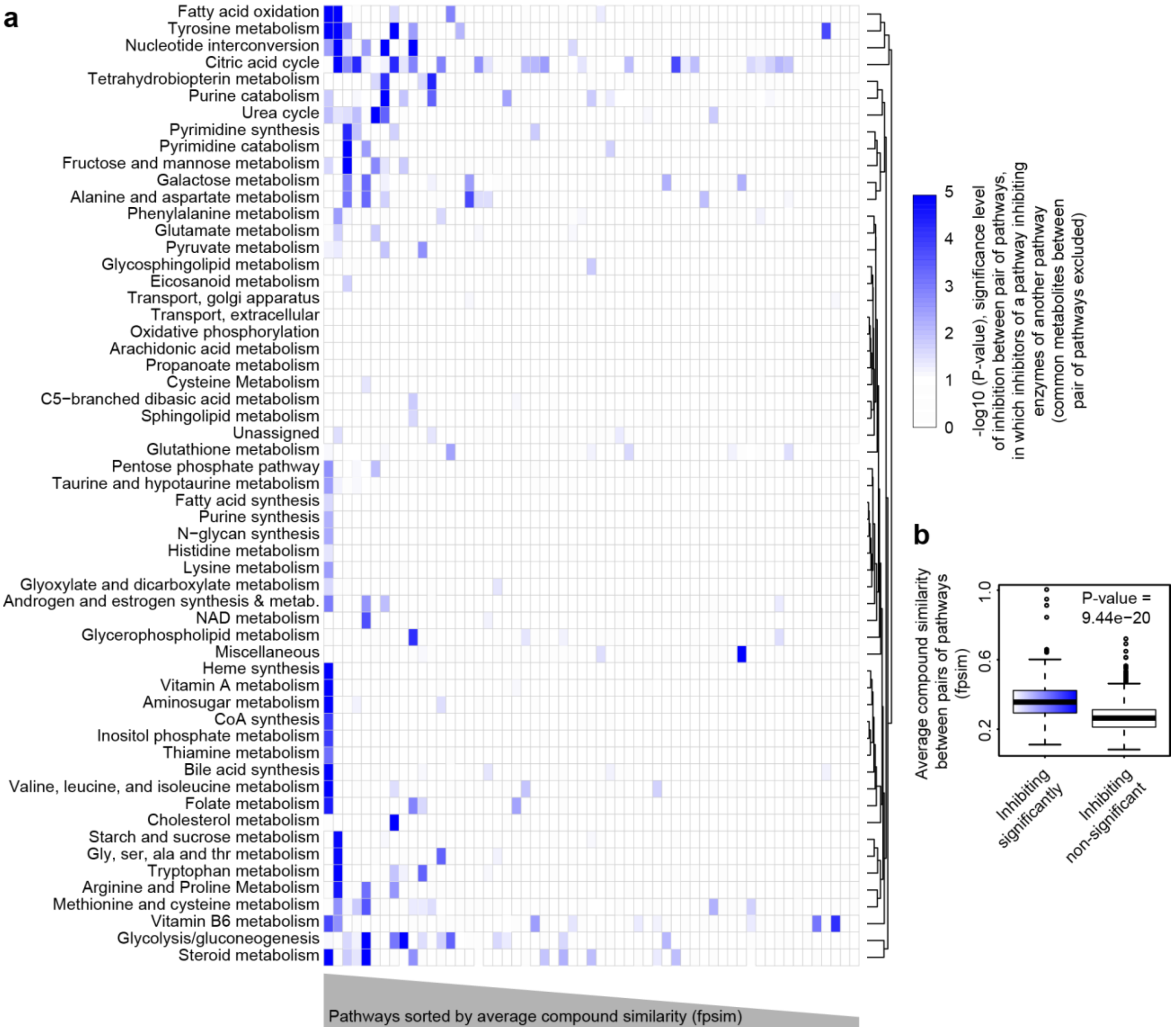

Supplementary Figure 5 (a) For every pathway (represented in rows), significance level of its inhibition to all other pathways (represented in columns) as shown in Supplementary Supplementary Figure 5, sorted by average compound similarity between the metabolites participating in the pathways. P-value calculated using hypergeometric test. (b) Average compound similarity between the metabolites participating in significantly cross-inhibiting pathways in trans is significant (P-value 9.44e-20, Welch Two Sample t-test), but no significant similarity is observed between metabolites participating in non-inhibiting pathways (excluding shared metabolites).

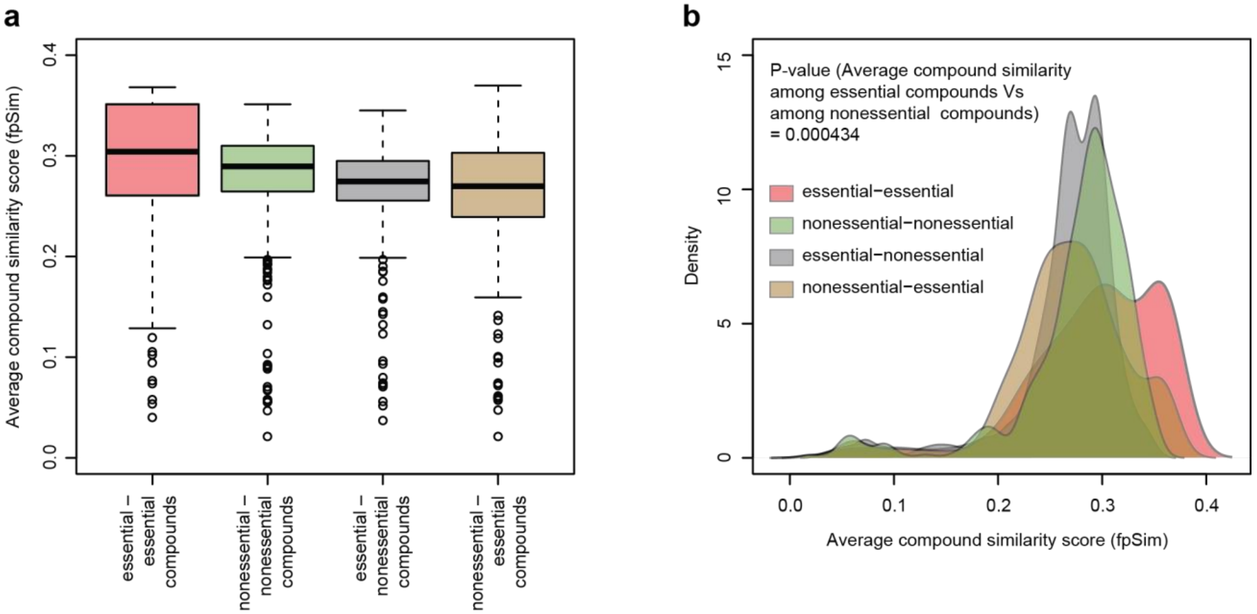

Supplementary Figure 6 Average compound similarity scores between essential - essential, nonessential - nonessential, essential - nonessential, and between nonessential - essential metabolites are shown in a (a) boxplot and in a (b) density plot. The average compound similarity between essential - essential metabolites is significantly higher than average compound similarity between all other cases, i.e. nonessential - nonessential, essential - nonessential, and between nonessential - essential compounds. P-value calculated using Welch Two Sample t-test.

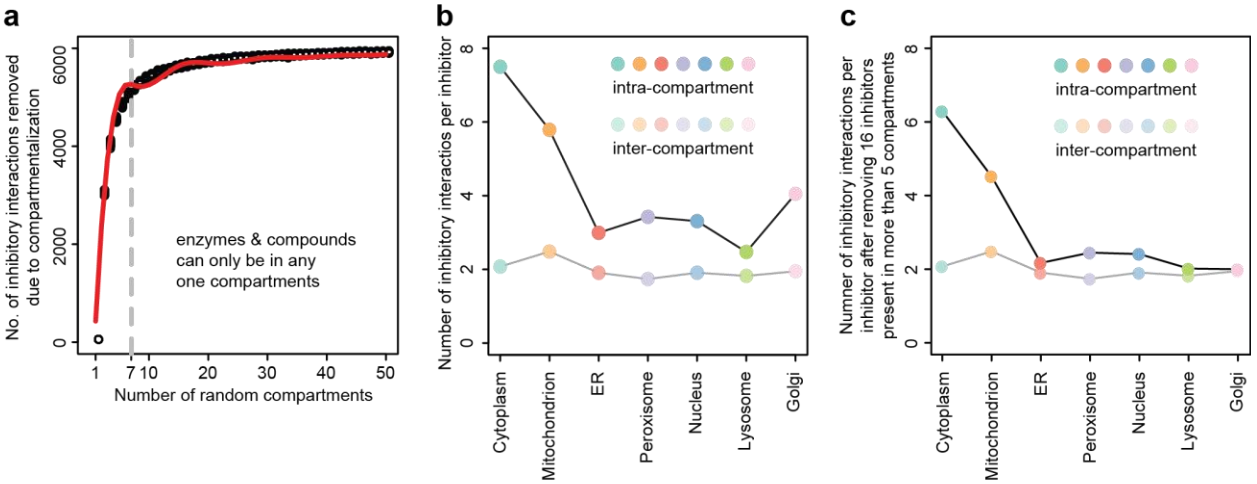

Supplementary Figure 7 (a) Computational simulation of the effect of compartmentalisation on the enzyme inhibition, based on randomly placing inhibitors and enzymes in any single random compartment. The red line represents *bs* spline fitting. (b) Average number of intra- and inter-compartmental inhibitory interactions per inhibitor. (c) Average number of intra- and inter-compartmental inhibitory interactions per inhibitor after removing the 16 ‘hub’ inhibitors (from Figure 4k) which are participating in the metabolism of more than 5 compartments.

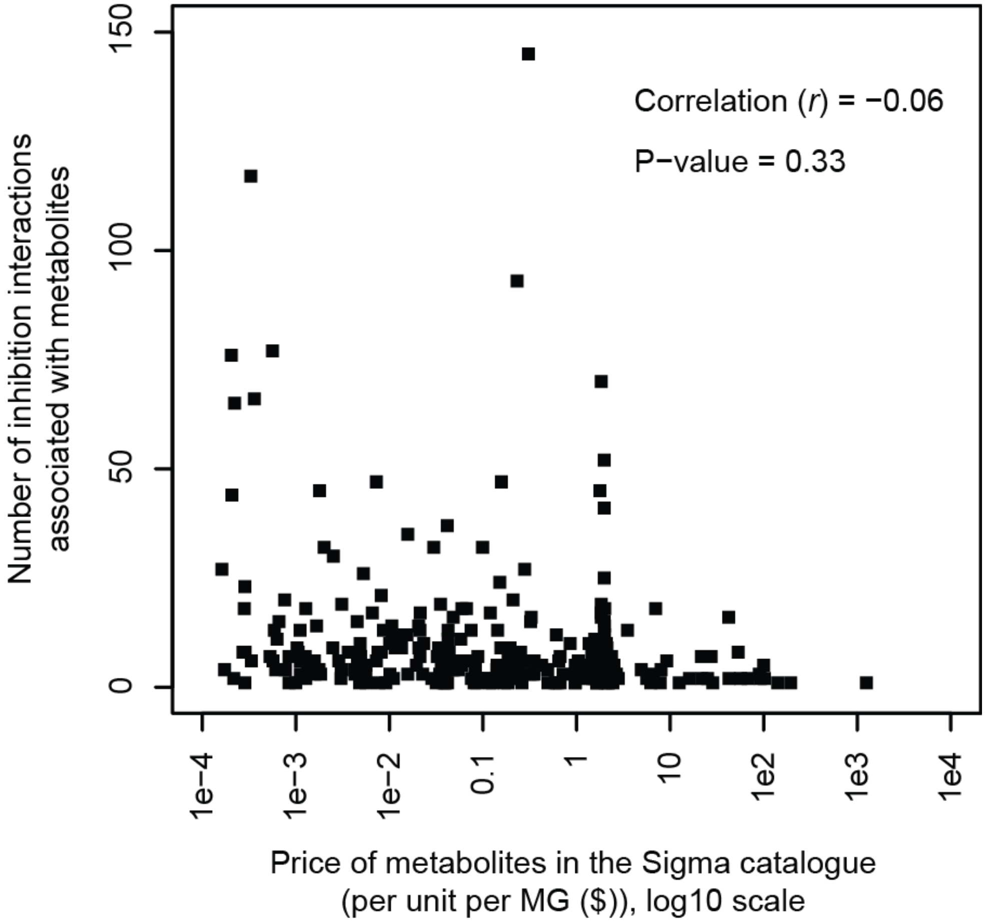

Supplementary Figure 8. No significant correlation between price of metabolite standard chemical (2016 Sigma Aldrich catalogue) and the number of inhibitory interactions associated with the chemical. Correlation test = Pearson's product-moment correlation.

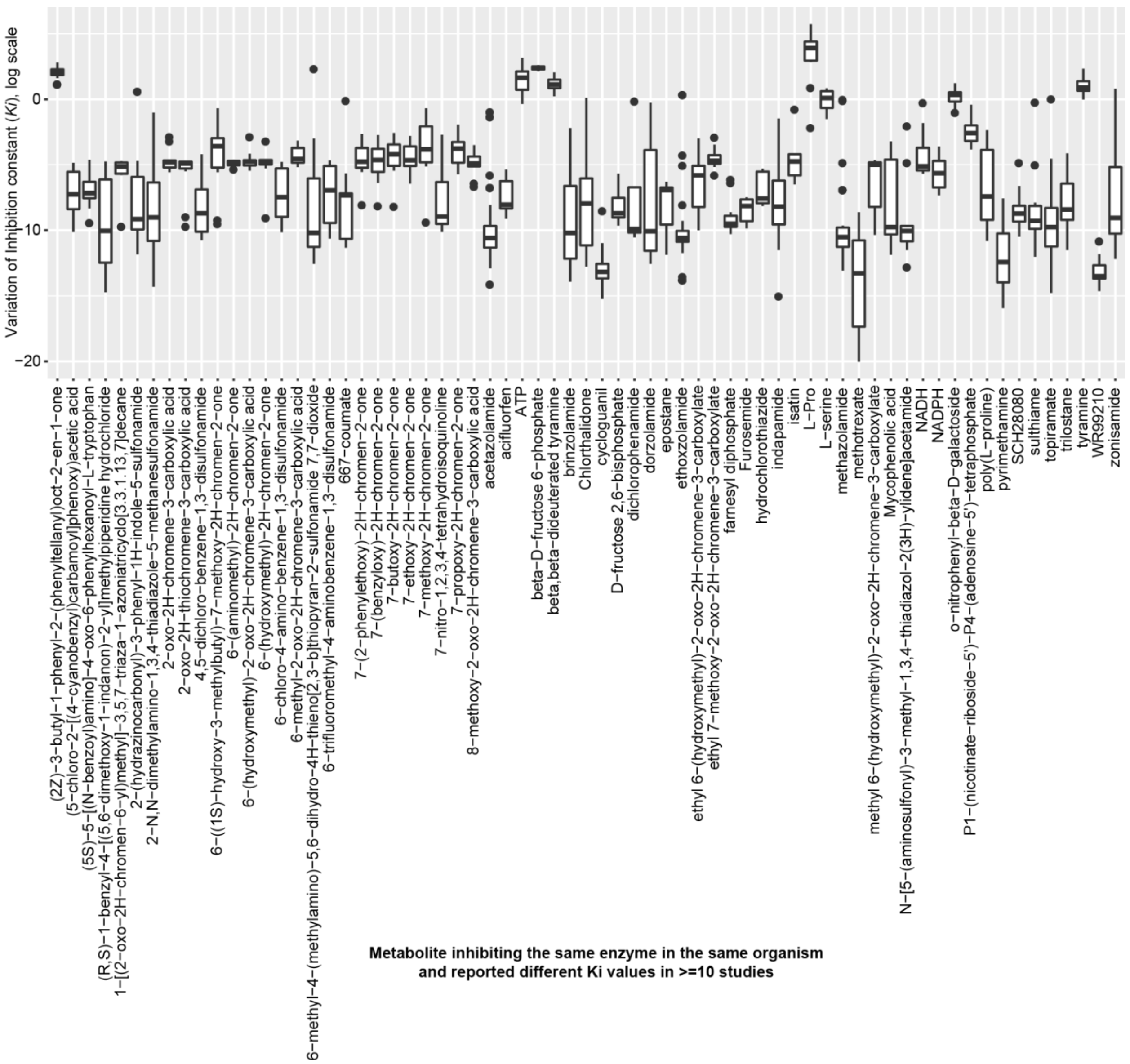

Supplementary Figure 9 Distribution of *Ki* values for inhibitor inhibiting the same enzyme in the same organism and reported in more than 10 studies. Ki values are highly divergent from study to study, and are given in log scale (Y axis).

Supplementary Tables

Supplementary Table 1: Enzyme coverage in the inhibition network

Enzyme Class	In Recon2 model	In inhibition network	Inhibitory interactions
Hydrolases	161	147	1389
Isomerases	39	32	188
Ligases	28	26	293
Lyases	66	49	497
Oxidoreductases	205	159	1658
Transferases	244	208	1964
Class not available	4		
Total	747	621	5989

Supplementary Table 2: Functional classification of inhibitors on the basis of HMDB database

<table>
<tr><th rowspan="2">HMDB Superclass</th><th colspan="3">In the inhibition network</th><th rowspan="2">In Recon 2</th><th rowspan="2">#Inhibitory interaction</th><th rowspan="2">Simplified group</th></tr>
<tr><th>Phosphorylated</th><th>Unphosphorylated</th><th>Total</th></tr>
<tr><td>Nucleosides, Nucleotides, and Analogues</td><td>70</td><td>14</td><td>84</td><td>102</td><td>1749</td><td></td></tr>
<tr><td>Organic Acids and Derivatives</td><td>2</td><td>41</td><td>43</td><td>69</td><td>537</td><td></td></tr>
<tr><td>Amino Acids, Peptides, and Analogues</td><td>3</td><td>82</td><td>85</td><td>176</td><td>613</td><td></td></tr>
<tr><td>Aliphatic Acyclic Compounds</td><td>8</td><td>32</td><td>40</td><td>65</td><td>541</td><td></td></tr>
<tr><td>Carbohydrates and Carbohydrate Conjugates</td><td>23</td><td>40</td><td>63</td><td>98</td><td>375</td><td></td></tr>
<tr><td>Lipids</td><td>47</td><td>131</td><td>178</td><td>610</td><td>810</td><td></td></tr>
<tr><td>Aromatic Heteropolycyclic Compounds</td><td>5</td><td>51</td><td>56</td><td rowspan="3">161</td><td rowspan="3">592</td><td rowspan="3">Aromatic Cyclic Compounds (total 105)</td></tr>
<tr><td>Aromatic Heteromonocyclic Compounds</td><td>3</td><td>12</td><td>15</td></tr>
<tr><td>Aromatic Homomonocyclic Compounds</td><td>0</td><td>34</td><td>34</td></tr>
<tr><td>Alkaloids and Derivatives</td><td>0</td><td>1</td><td>1</td><td rowspan="5">376</td><td rowspan="5">772</td><td rowspan="5">Other metabolites (total 84)</td></tr>
<tr><td>Aliphatic Homomonocyclic Compounds</td><td>8</td><td>1</td><td>9</td></tr>
<tr><td>Aliphatic Heteropolycyclic Compounds</td><td>0</td><td>2</td><td>2</td></tr>
<tr><td>Benzenoids</td><td>0</td><td>1</td><td>1</td></tr>
<tr><td>Homogeneous Non-metal</td><td>4</td><td>12</td><td>16</td></tr>
</table>

Compounds						
Organophosphorus Compounds	6	0	6			
Organooxygen Compounds	2	0	2			
Aliphatic Heteromonocyclic Compounds	0	7	7			
Missing HMDB ID	16	24	40			

Supplementary Table 3: Number of inhibitions from different functional groups inhibiting different enzyme classes.

	Hydrolases	Oxidoreductases	Transferases	Lyases	Ligases	Isomerases
Nucleosides, Nucleotides, and Analogues	356	369	777	110	104	33
Others	235	167	217	76	41	36
Organic Acids and Derivatives	71	243	112	61	36	14
Amino Acids, Peptides, and Analogues	145	157	208	71	25	7
Aliphatic Acyclic Compounds	155	171	135	55	16	9
Carbohydrates and Carbohydrate Conjugates	141	35	117	26	8	48
Lipids	178	267	249	36	45	35
Aromatic Cyclic Compounds	108	249	149	62	18	6

Supplementary Table 4: Significance of inhibition from functional groups inhibiting enzyme classes.

	Hydrolases	Oxidoreductases	Transferases	Lyases	Ligases	Isomerases
Nucleosides, Nucleotides, and Analogues	0.99	1	4.8e-34	0.99	0.01	0.99
Others	4.2e-7	0.99	0.99	0.057	0.30	8.6e-3
Organic Acids and Derivatives	1	5.3e-20	1	6e-3	3.1e-02	0.80
Amino Acids, Peptides, and Analogues	0.40	0.89	0.28	1.8e-3	0.86	0.99
Aliphatic Acyclic Compounds	1.2e-3	2e-2	0.99	0.06	0.99	0.99
Carbohydrates and Carbohydrate Conjugates	6.7e-11	1	0.77	0.86	0.99	5.9e-18
Lipids	0.82	2.3e-4	0.92	0.99	0.196	2.9e-2
Aromatic Cyclic Compounds	0.99	1.5e-15	0.99	2.9e-2	0.99	0.99

Supplementary Table 5: FDR of inhibition from from functional groups inhibiting enzyme classes.

	Hydrolases	Oxidoreductases	Transferases	Lyases	Ligases	Isomerases
Nucleosides, Nucleotides, and Analogues	0.98	1	0	1	0.01	1
Others	0.01	0.97	0.99	0.06	0.22	0.05
Organic Acids and Derivatives	1	0	1	0	0.05	0.72
Amino Acids, Peptides, and Analogues	0.36	0.66	0.37	0	0.79	1
Aliphatic Acyclic Compounds	0.03	0.18	0.97	0.07	0.94	0.97
Carbohydrates and Carbohydrate Conjugates	0	1	0.57	0.83	0.98	0
Lipids	0.66	0.05	0.71	1	0.27	0.04
Aromatic Cyclic Compounds	0.99	0	0.99	0.07	0.96	1

Supplementary References

1. Cao, D.-S., Xiao, N., Xu, Q.-S. & Chen, A. F. Rcpi: R/Bioconductor package to generate various descriptors of proteins, compounds and their interactions. *Bioinformatics* 31, 279–281 (2015).
2. Cao, Y., Charisi, A., Cheng, L.-C., Jiang, T. & Girke, T. ChemmineR: a compound mining framework for R. *Bioinformatics* 24, 1733–1734 (2008).
3. Wang, Y., Backman, T. W. H., Horan, K. & Girke, T. fmcsR: mismatch tolerant maximum common substructure searching in R. *Bioinformatics* 29, 2792–2794 (2013).
4. Cao, Y., Charisi, A., Cheng, L.-C., Jiang, T. & Girke, T. ChemmineR: a compound mining framework for R. *Bioinformatics* 24, 1733–1734 (2008).
